# Physics-Informed CNN-BiGRU Model for Downhole Weight-on-Bit Prediction Using Surface Measurement-While-Drilling Sensor Data in Horizontal Wells

**DOI:** 10.3390/s26154747

**Published:** 2026-07-26

**Authors:** Zebing Wu, Lianghui Song, Jun Xu, Jian Chen, Tianci Wang, Qinglin Wang, Siqi Wang

**Affiliations:** 1 Mechanical Engineering College, Xi’an Petroleum University, Xi’an 710065, China; 24212040728@stumail.xsyu.edu.cn (T.W.); 24211040617@stumail.xsyu.edu.cn (Q.W.); 24212040714@stumail.xsyu.edu.cn (S.W.); 2 Sinopec SJ Petroleum Machinery Co., Jingzhou 434024, China; xuj.oset@sinopec.com (J.X.); chenj808.oset@sinopec.com (J.C.)

**Keywords:** horizontal well, DWOB, surface sensor data and downhole MWD measurements, physics-informed neural network (PINN), CNN-BiGRU, WOA, drill-string frictional attenuation

## Abstract

**Highlights:**

**What are the main findings?**

- A WOA-optimized physics-informed CNN-BiGRU model was developed for DWOB prediction.
- Drill-string frictional attenuation was incorporated into the loss function.

**What are the implications of the main findings?**

- DWOB can be estimated from surface sensor data without direct downhole measurements.
- The method supports drilling parameter optimization and intelligent drilling control.

**Abstract:**

Accurate estimation of downhole weight on bit (DWOB) is important for drilling parameter optimization and safe drilling in extended-reach horizontal wells. However, DWOB is difficult to obtain continuously because surface weight on bit (SWOB) is attenuated by drill-string friction, wellbore trajectory, and borehole-wall contact. To address this problem, a physics-informed convolutional neural network combined with bidirectional gated recurrent unit (CNN-BiGRU) prediction model optimized by the whale optimization algorithm (WOA) is proposed using surface sensor data and downhole measurement-while-drilling (MWD) measurements. The model first uses a one-dimensional CNN to extract local features from drilling parameters along the measured-depth direction and then employs a BiGRU to capture depth-series dependencies. Meanwhile, a drill-string frictional attenuation relationship is embedded into the loss function as a physical prior, and WOA is used to optimize network hyperparameters and the physics-constrained weight. Field data from a horizontal well were used for validation. The proposed model achieved a coefficient of determination (R^2^)of 0.9245 on the test set, with root mean square error (RMSE), mean absolute error (MAE), and mean relative error (MRE) values of 0.1856, 0.1135, and 0.0124, respectively. The results demonstrate that the proposed data–physics hybrid framework improves the accuracy and physical consistency of DWOB prediction.

## 1. Introduction

With the continuous extension of oil and gas exploration and development toward deep, ultra-deep, and unconventional reservoirs, extended-reach horizontal wells have played an increasingly important role in improving drilling productivity and reducing development costs [1,2]. During horizontal well drilling, DWOB is a key parameter affecting rock-breaking efficiency, rate of penetration (ROP), and drilling safety. However, continuous direct measurement of DWOB is often limited by downhole tool availability, measurement cost, data transmission conditions, and the harsh drilling environment. In contrast, surface sensor data, such as hook load, SWOB, standpipe pressure (SPP), torque, ROP, and well trajectory parameters, can be continuously acquired during drilling operations. These sensor measurements provide an important data basis for indirectly estimating the actual DWOB state.

Nevertheless, during extended-reach horizontal drilling, the drill string is jointly affected by the wellbore trajectory, drill-string weight, borehole-wall contact, and complex downhole operating conditions. As a result, extensive contact occurs between the drill string and the borehole wall in the horizontal section, leading to a significant increase in frictional resistance along the wellbore. Owing to the cumulative friction effect, the SWOB is substantially attenuated during transmission to the bit, resulting in a typical “WOB hold-up” phenomenon [3]. Consequently, SWOB and other surface sensor parameters cannot directly represent the actual DWOB. Insufficient DWOB reduces rock-breaking efficiency and prolongs the drilling cycle, whereas blindly increasing the SWOB may induce drill-string buckling, stick-slip vibration, fatigue damage, or even downhole tool failure [4,5]. Therefore, developing an accurate, physically interpretable, and sensor-data-driven method for DWOB prediction is important for drilling parameter optimization and safe drilling control in horizontal wells.

For the transmission of WOB and the calculation of drill-string frictional drag, previous studies have mainly developed physics-based models from the perspectives of drill-string force analysis and torque-and-drag modeling. Chen et al. [6] incorporated factors such as drill-string friction and well inclination into WOB transmission analysis and evaluated the influence of downhole power tools on DWOB. Li Wenfei et al. [7] established an improved longitudinal–transverse bending beam model considering virtual contact points and verified its computational accuracy under casing running, rotary drilling, sliding drilling, and compound drilling conditions. Wu Zebing et al. [8] combined the Johancsik model with the Aadnoy three-dimensional model, considered the influence of pipe-string stiffness, and developed a computational program for DWOB estimation. These physics-based models can reveal the attenuation mechanism of WOB transmission from the perspectives of wellbore trajectory, drill-string loading, and frictional contact, and they have clear mechanical foundations and good interpretability. However, such models generally rely on idealized assumptions and fixed parameters, and are more suitable for static calculation or post-operation analysis. They may have difficulty capturing, in real time, the nonlinear responses caused by formation changes, wellbore trajectory variations, and fluctuations in drilling conditions [9,10]. Consequently, relying solely on traditional mechanical models cannot meet the requirements of real-time DWOB prediction and dynamic control in field drilling operations.

In recent years, with the development of MWD technology and artificial intelligence methods, machine learning and deep learning models have been increasingly applied to drilling parameter prediction, operating condition identification, and ROP optimization [11,12]. Compared with traditional physics-based models, data-driven methods can automatically extract complex nonlinear features from large amounts of field data, demonstrating strong fitting capability and potential for real-time prediction. For example, Liu et al. [13] developed an artificial-neural-network-based model for drilling ROP prediction, while Samin et al. [14] used machine-learning methods to forecast bottom-hole pressure under multiphase-flow conditions. Zhang et al. [15] developed an ensemble-learning-based intelligent early-warning model for lost circulation. In addition, Al-Rubaii et al. [16] developed an artificial-intelligence-based model for real-time prediction of drilling fluid density and equivalent circulating density, demonstrating the potential of sensor-based drilling parameters for real-time drilling monitoring and decision support. Yang et al. [17] investigated multi-sensor combined measurement-while-drilling and showed that adaptive filtering and sensor-data fusion can improve the robustness of MWD measurements under downhole interference conditions. Cheng et al. [18] further used torque-related while-drilling information for fine detection of strata information, indicating that drilling sensor signals can reflect formation and operating-condition variations during drilling. These studies suggest that surface sensor data and downhole MWD measurements contain valuable information for drilling process monitoring, intelligent parameter prediction, and drilling decision-making.

For drilling-parameter prediction and DWOB prediction, Khosravanian et al. [19] constructed a fuzzy inference-based WOB prediction model using parameters such as permeability, drilling fluid density, and standpipe pressure as inputs. Bai et al. [20] applied just-in-time learning to real-time drilling torque prediction ahead of the bit, considered the similarity between current and historical drilling scenarios, and developed a local-model-based forecasting framework. Zhang et al. [21] proposed a DWOB prediction method based on a convolutional neural network-bidirectional long short-term memory (CNN-Bi-LSTM) network optimized by the dual-strategy collaborative search optimization algorithm. In addition, deep sequence models and hybrid neural-network architectures have shown good potential for depth-dependent subsurface data analysis. For example, Zhao et al. [22] combined CNN, BiLSTM, and temporal convolutional network (TCN) to extract local features, bidirectional sequential dependencies, and long-range depth-series information from logging data for lithology identification. These studies indicate that CNN, recurrent neural networks (RNN), and their improved variants have shown good performance in local feature extraction and temporal dependency modeling, providing new technical approaches for complex drilling parameter prediction. However, field surface sensor data and downhole MWD measurements usually contain strong noise, large fluctuations, and non-stationary operating conditions. Purely data-driven models lack explicit physical boundary constraints and are therefore prone to overfitting, prediction lag, and nonphysical outputs that violate drill-string mechanical laws [23,24], thereby reducing their generalization capability and engineering reliability under complex drilling conditions. Therefore, for DWOB prediction in horizontal wells, it is necessary to integrate data-driven sequence modeling with drill-string mechanical constraints so that the prediction results can better reflect both nonlinear sensor-data relationships and the physical attenuation mechanism of WOB transmission.

To improve the interpretability and physical consistency of data-driven drilling prediction models, hybrid physics–machine learning methods have recently attracted increasing attention. Jiao et al. [25] developed hybrid physics–machine learning models for rate-of-penetration prediction in the Halahatang oil field and showed that combining physical modeling with machine learning can improve prediction accuracy while preserving interpretability. This provides useful evidence that incorporating physical knowledge into data-driven drilling models is a feasible strategy for complex drilling parameter prediction.

PINN provides a new approach to integrating physical constraints into neural-network training. The PINN proposed by Raissi et al. embeds governing equations or physical constraints into the loss function of neural networks, enabling the model to learn data features while being constrained by physical laws, thereby balancing data-fitting accuracy and physical consistency [26]. For DWOB prediction in horizontal wells, introducing the drill-string frictional attenuation equation into a deep learning model as a mechanical prior can effectively limit nonphysical deviations in model outputs and improve the interpretability and reliability of the prediction results. However, during the training of PINN, gradient-scale differences often exist between the data loss and the physics loss. If the physics-constrained weight and network hyperparameters are determined solely by empirical settings, the model may suffer from unstable convergence or become trapped in local optima [27]. Therefore, an adaptive optimization strategy is needed to jointly optimize the structural parameters of the deep network and the physics-constrained weight, thereby improving the overall predictive performance of the model.

Based on the above considerations, this study develops a WOA-optimized physics-informed CNN-BiGRU (WOA-PINN-CNN-BiGRU) model for DWOB prediction using surface sensor data in horizontal wells. The main purpose is not to design a new downhole sensor, but to establish a sensor-data-driven estimation method for DWOB when continuous direct downhole measurements are limited or difficult to obtain. The main contributions of this study are summarized as follows. First, a DWOB estimation framework is constructed using surface sensor data, providing a feasible approach for inferring the actual bit load from surface-measured drilling responses. Second, a drill-string frictional attenuation relationship is embedded into the neural network loss function as a physical prior, thereby improving the mechanical consistency and interpretability of the prediction results. Third, CNN-BiGRU architecture is adopted to extract local feature patterns and bidirectional depth-series dependencies from multivariate surface sensor parameter sequences. Finally, WOA is introduced to optimize the network hyperparameters and the physics-constrained weight, and the effectiveness of each module is evaluated through benchmark comparisons and ablation experiments using field data from a horizontal well. The results provide a physically consistent and sensor-data-driven prediction method for DWOB estimation, offering a potential basis for drilling parameter optimization and future intelligent drilling control after further field validation.

## 2. Materials and Methods

### 2.1. Overall Framework

To achieve accurate DWOB prediction in horizontal wells using surface sensor data and downhole MWD measurements, this study develops a WOA-PINN-CNN-BiGRU hybrid prediction model. The proposed method integrates field sensor data preprocessing, feature selection, depth-series feature extraction, physics-informed constraint embedding, and adaptive hyperparameter optimization. The overall objective is to estimate DWOB from surface-measured drilling parameters while improving both prediction accuracy and physical consistency.

**Figure 1:**
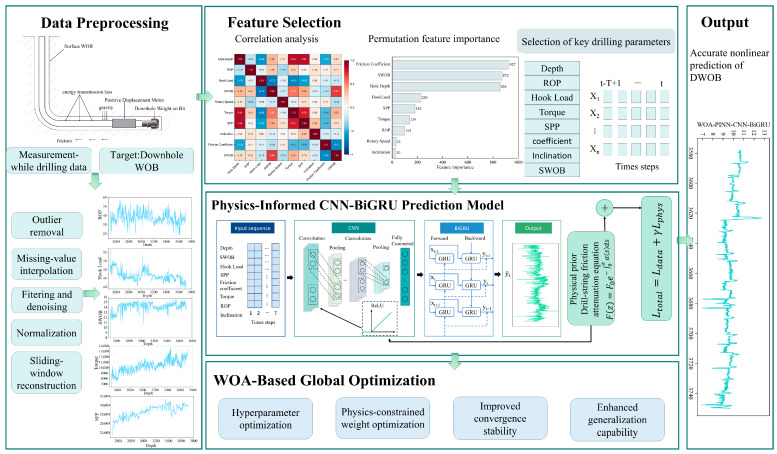
Overall workflow of the WOA-PINN-CNN-BiGRU model for DWOB prediction using surface sensor data.

Overall, the proposed model framework consists of five components: data preprocessing, feature selection, the physics-informed CNN-BiGRU prediction model, WOA-based global optimization, and prediction output. First, the raw sensor data are processed through outlier removal, missing-value interpolation, filtering and denoising, normalization, and sliding-window reconstruction to generate depth-series samples suitable for neural network input. Second, correlation analysis and permutation feature importance analysis are combined to identify the key drilling parameters affecting DWOB. Then, the CNN-BiGRU network is employed to extract local features and bidirectional depth-series dependencies from the drilling parameter sequences, while the drill-string frictional attenuation relationship is embedded into the loss function as a physical prior to construct a physics-informed prediction model. Subsequently, WOA is introduced to perform global optimization of the network hyperparameters and the physics-constrained weight, thereby improving the prediction accuracy, convergence stability, and generalization capability of the model under the tested field-data conditions. Finally, the model outputs the predicted DWOB, providing a basis for model evaluation and drilling parameter optimization. The overall workflow of the proposed model is illustrated in Figure 1.

### 2.2. DWOB Transmission Mechanism and Physics-Constrained Model in Horizontal Wells

DWOB is the effective axial load acting at the bit–rock contact interface and is an important factor affecting rock-breaking efficiency and ROP. In the horizontal section, the axial load transmission of the drill string is not an ideal linear process because it is affected by the gravity component, wellbore curvature, and borehole wall constraints. Instead, it is accompanied by significant contact-induced frictional losses. As the measured depth increases, the contact region between the drill string and the borehole wall gradually expands, and the frictional resistance accumulates along the wellbore trajectory, causing the effective WOB transmitted to the bit to be lower than the SWOB. Therefore, DWOB can be regarded as the attenuated result of SWOB under drill-string–borehole-wall friction, and its variation is closely related to the wellbore trajectory, friction coefficient, well inclination, and drill-string loading state. From the perspective of drill-string mechanics, WOB transmission loss is mainly affected by factors such as wellbore trajectory, well inclination, friction coefficient, drill-string stiffness, and borehole-wall contact state. The classical Johancsik model, based on infinitesimal force analysis of the drill string, established a mechanical relationship for the attenuation of axial force along the measured depth in directional wells, providing a theoretical basis for DWOB estimation and torque-and-drag analysis [28]. Based on this type of drill-string frictional mechanics model, this study introduces the WOB attenuation equation into the neural network training process as a physical prior to constrain the model output, enabling the predicted DWOB to satisfy basic mechanical laws while fitting the data.

**Figure 2:**
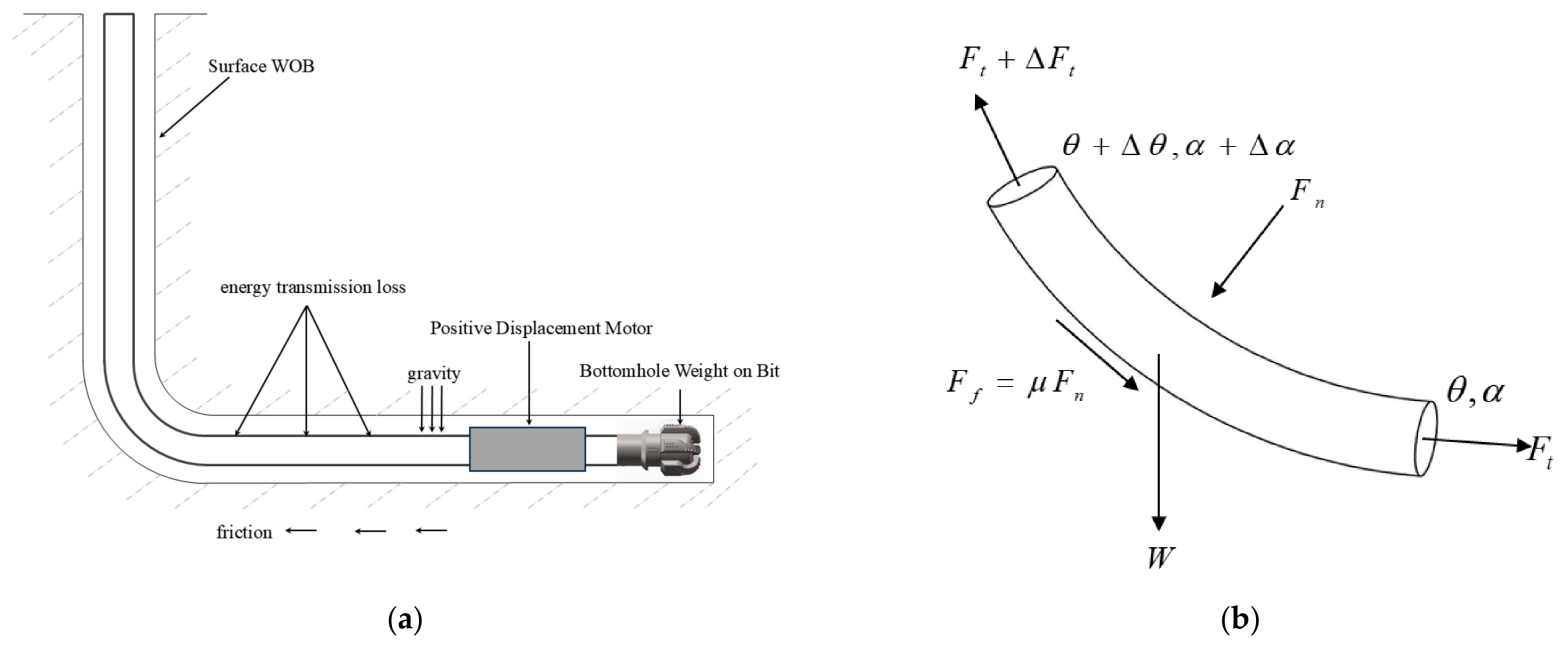
Schematic diagram of WOB transmission attenuation and infinitesimal force analysis of the drill string: (**a**) WOB transmission loss in a horizontal well; (**b**) force analysis of a drill string micro-element.

Figure 2 illustrates the attenuation mechanism of WOB transmission in a horizontal well and the force relationship of a drill-string micro-element. Specifically, Figure 2a on the left describes the process by which the effective DWOB decreases during the transmission of SWOB along the wellbore trajectory, owing to drill-string–borehole-wall contact friction, the gravity component, and energy transmission loss. Figure 2b on the right presents the force analysis of a drill-string micro-element under the combined effects of axial force, normal contact force, friction force, gravity, and wellbore curvature. Therefore, this schematic explains the mechanical origin of DWOB attenuation from both the overall load transmission process and the local micro-element force balance.

Under rotary drilling conditions, the SWOB is gradually attenuated during its transmission to the bit owing to the combined effects of drill-string–borehole-wall contact, frictional resistance, wellbore trajectory, and the gravity component. Considering that the complete dynamic contact process of the drill string is difficult to describe using a single analytical equation under field drilling conditions, this study adopts a simplified drill-string frictional attenuation relationship as a physics-based prior rather than a strict full-order governing equation. The physics-based reference value of DWOB can be expressed as:

(1) $${DWOB}^{phys}=SWOBe^{-\mu_{s}\eta_{b}}$$

where ${DWOB}^{phys}$ denotes the physics-based reference value of downhole weight on bit, SWOB denotes the surface weight on bit, $\mu_{s}$ is the equivalent friction coefficient under rotary drilling conditions, and $\eta_{b}$ is the inclination-related equivalent contact attenuation coefficient.

In this study, the field-measured DWOB and the physics-based reference value of DWOB are clearly distinguished. The field-measured DWOB is used as the supervised learning label for calculating the data-fitting loss. In contrast, the physics-based reference value of DWOB is not directly obtained from field measurements, but is calculated from the simplified drill-string frictional attenuation equation. This reference value is used only to construct the physics-informed loss term and to provide a mechanical constraint during model training. In the context of DWOB prediction, physical consistency means that the predicted DWOB should not only fit the MWD measured DWOB data, but also follow the basic mechanical attenuation trend of WOB transmission caused by drill-string–borehole-wall contact, frictional resistance, wellbore trajectory, and effective contact length.

This equation does not aim to fully replace detailed torque-and-drag analysis or dynamic drill-string contact modeling. Instead, it provides a simplified physical reference that reflects the general attenuation trend of WOB transmission along the wellbore. By embedding this attenuation relationship into the loss function, the neural network prediction is constrained to remain consistent with the basic mechanical behavior of drill-string frictional attenuation while still retaining the nonlinear fitting capability of data-driven learning.

### 2.3. Data Preprocessing and Depth-Series Sample Construction

Field surface sensor data and downhole MWD measurements from an actual horizontal well in western Canada were used for model training, validation, and testing. The measured DWOB labels were acquired using a commercial downhole MWD tool installed in the bottom-hole assembly. This tool is capable of acquiring and transmitting downhole drilling measurements, including DWOB, during the drilling process. The available field dataset used in this study was organized according to measured depth, in which the surface parameters and the corresponding measured DWOB labels were matched at the same measured-depth positions. Therefore, the data synchronization in this study was depth-based rather than time-based. The exact raw acquisition frequency of the DWOB measurements was not included in the dataset available to the authors. Records with missing DWOB labels, abnormal measurements, or inconsistent depth information were removed before model training.

Owing to complex downhole operating conditions, sensor measurement errors, data transmission noise, and local operational disturbances, the raw sensor data may contain unreasonable outliers, missing values, and high-frequency fluctuations. To improve data quality while avoiding information leakage, all preprocessing procedures were carried out under a depth-ordered data-processing strategy.

First, unreasonable outliers were removed according to predefined engineering and sensor-validity ranges of the drilling parameters. Specifically, data points that far exceeded the physically reasonable operating ranges of parameters such as SWOB, hook load, SPP, torque, ROP, and inclination angle were regarded as abnormal records caused by sensor errors, communication faults, or abnormal operating states and were removed. This outlier removal criterion was based on engineering feasibility and instrument validity rather than statistical information from the validation or test sets.

Second, missing values in the surface sensor data and downhole MWD measurements sequences were reconstructed by interpolation to maintain the continuity of the measured-depth sequence. The interpolation process was performed without using any target information from the validation or test sets. Subsequently, a moving average filtering method was applied to suppress high-frequency fluctuations in the input sensor signals while preserving the overall variation trends of the original data. The filtering operation was conducted along the measured-depth direction and did not disturb the sequential order of the drilling data.

**Figure 3:**
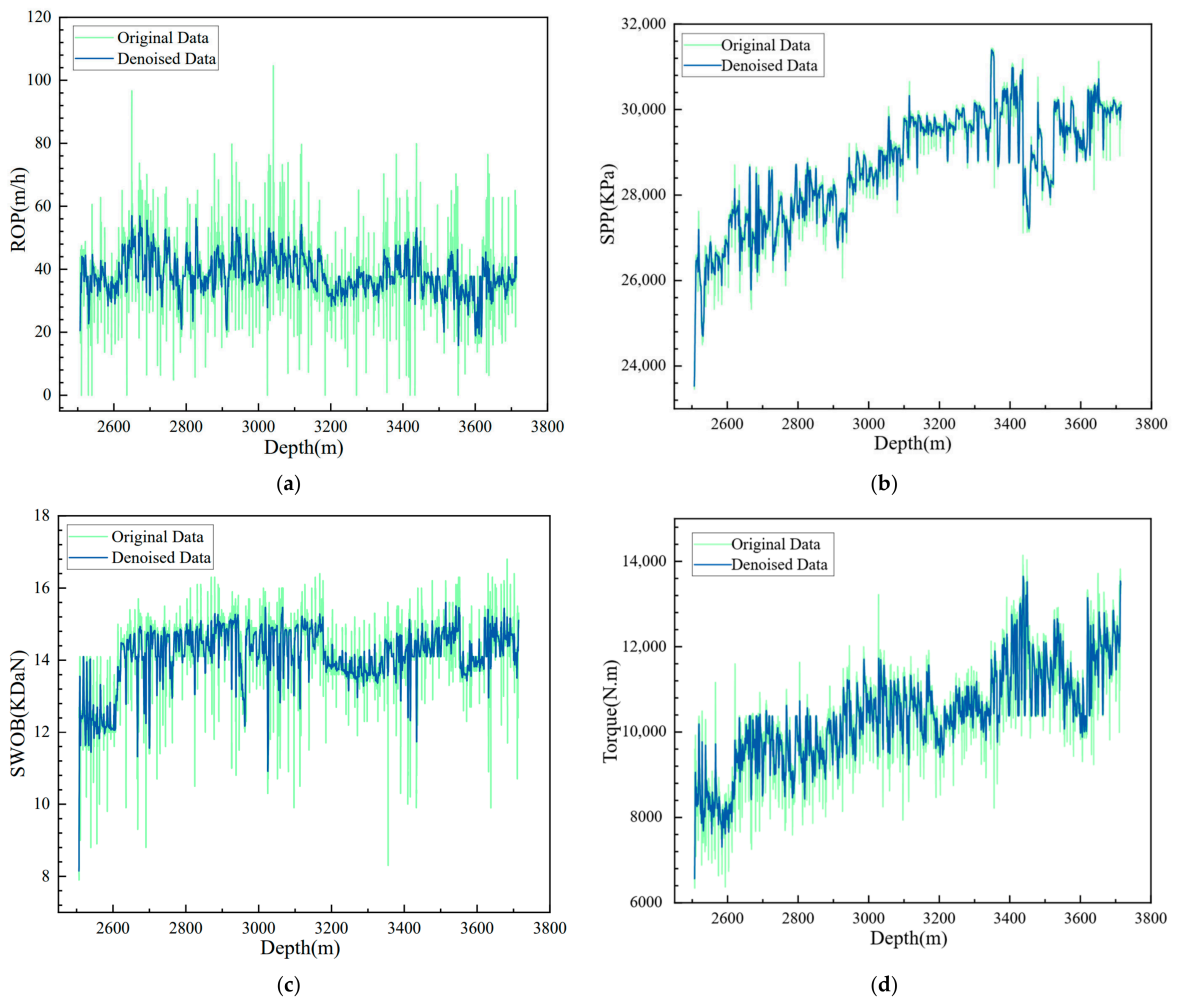
Comparison of representative sensor parameters before and after denoising for Well A: (**a**) ROP; (**b**) SPP; (**c**) SWOB; (**d**) Torque.

To demonstrate the smoothing effect of the preprocessing method on drilling parameter signals, representative sensor parameters from Well A were selected for comparison before and after denoising, as shown in Figure 3. Specifically, Figure 3a in the upper right represents the ROP, Figure 3b in the upper left represents the SPP, Figure 3c in the lower right represents the SWOB, and Figure 3d in the lower left represents the torque. It can be observed that the moving average filtering method suppresses high-frequency fluctuations and local abnormal disturbances while preserving the overall variation trend of the original signals, thereby providing more stable input data for subsequent model training.

After preprocessing and sliding-window reconstruction, a total of 9957 valid samples were obtained from the horizontal well. Considering the strong sequential correlation of sensor data along the measured-depth direction, the dataset was divided strictly according to depth order rather than by random sampling. Specifically, 6970 samples, accounting for approximately 70% of the dataset, were used for model training; 996 samples, accounting for approximately 10%, were used for validation; and the remaining 1991 samples, accounting for approximately 20%, were used for final testing.

The validation set was used for WOA fitness evaluation, learning-rate adjustment, early stopping, and model selection, whereas the test set was used only for final performance evaluation. This partitioning strategy avoids data leakage caused by random splitting and is more consistent with the engineering scenario in which data from drilled sections are used to predict DWOB variations in subsequent well sections.

To further prevent information leakage, the normalization parameters, including the minimum and maximum values of each input variable, were calculated only from the training set. The same normalization parameters were then applied to the validation and test sets. Therefore, no statistical information from the validation or test sets was used to determine the normalization parameters, thereby improving the reliability of the model evaluation. The Max–Min normalization formula is given as follows:

(2) $$\tilde{x}=\frac{x-x_{min}}{x_{max}-x_{min}}$$

In Equation (2): $\tilde{x}$ is the normalized feature; *x* is the original feature; $x_{min}$ is the minimum value of the feature; and $x_{max}$ is the maximum value of the feature. This scaling ensures that all variables contribute equally to the model training process and improves the convergence efficiency of the deep learning algorithm.

Considering the strong continuity and sequential correlation of the drilling process along the measured-depth direction, a sliding-window method was adopted to reconstruct the continuous sensor data into depth-series input samples. This allows the model to utilize local sequential correlations between adjacent depth points and more accurately characterize the nonlinear variation in DWOB with measured depth.

### 2.4. Input Feature Selection

To improve the effectiveness of the input variables and reduce the influence of redundant or weakly relevant features during model training, Spearman correlation coefficient analysis and permutation feature importance analysis were jointly used to screen the candidate drilling parameters. The Spearman correlation coefficient was used to evaluate the monotonic relationships among variables, while permutation feature importance was used to quantify the contribution of each candidate parameter to DWOB prediction by perturbing individual features and observing the resulting change in model performance [29].

The candidate variables included hole depth, ROP, hook load, SWOB, rotary speed, torque, SPP, inclination angle, and friction coefficient. DWOB was used as the target output for correlation and importance analysis, but it was not used as an input feature.

The friction coefficient was not directly measured by MWD tools, but was obtained as an equivalent engineering parameter through torque-and-drag analysis. The equivalent friction coefficient was estimated using the torque-and-drag calculation method described in Ref. [8], which integrates the Johancsik model and the Aadnoy 3D model while considering the effect of drill-string stiffness. Off-bottom rotating data, collected when the bit was not in contact with the formation and the drill string was rotating during the downward operation, were used for calibration. An initial friction coefficient was assumed, and the corresponding surface hook load was iteratively calculated and compared with the measured hook load from field data. The friction coefficient was adjusted until the calculated hook load agreed well with the measured hook load within an acceptable tolerance. Therefore, the resulting friction coefficient was used as a model-calibrated equivalent friction coefficient rather than a raw MWD measurement.

**Figure 4:**
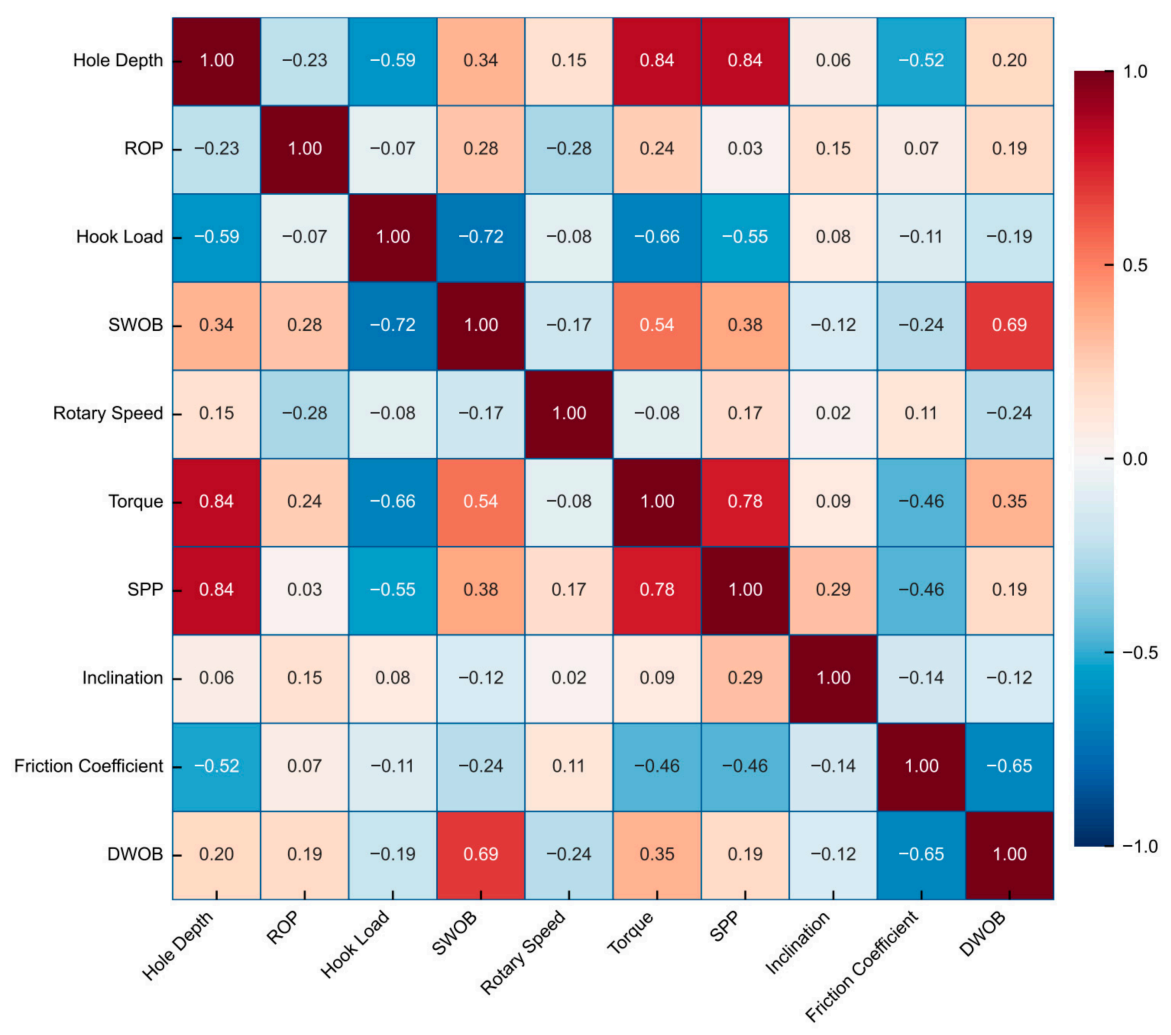
Correlation analysis of candidate drilling parameters and DWOB.

**Figure 5:**
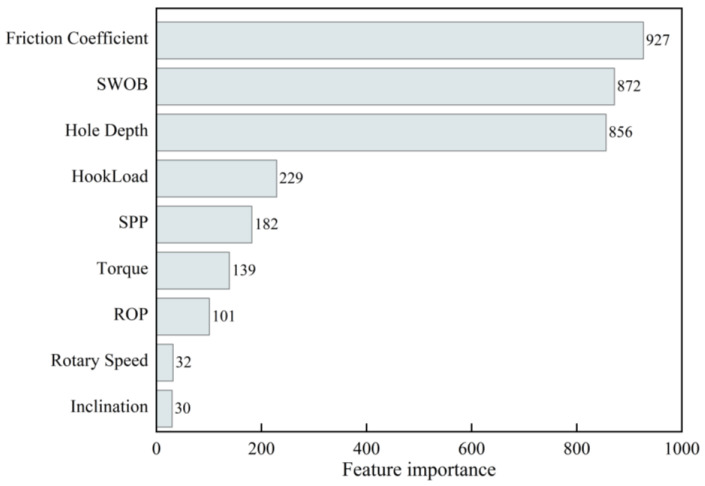
Permutation feature importance analysis of candidate input parameters.

As shown in Figure 4, some variables exhibit relatively strong correlations. For example, hole depth is highly correlated with torque and standpipe pressure, and SWOB also shows a strong correlation with hook load. This indicates that certain drilling parameters contain partially overlapping information. However, these parameters represent different physical aspects of the drilling process, such as axial loading, hydraulic response, drill-string rotation resistance, and wellbore trajectory effects. Therefore, feature selection in this study was not based solely on statistical correlation, but also considered the physical relevance of each parameter to WOB transmission. The permutation feature importance results shown in Figure 5 further indicate that friction coefficient, SWOB, and hole depth make the largest contributions to DWOB prediction. This is consistent with the physical mechanism of WOB transmission, because the DWOB is strongly affected by the surface-applied axial load, frictional attenuation, and cumulative wellbore contact along the measured depth. Hook load, SPP, torque, and ROP also contribute to the prediction by reflecting the drill-string loading state, hydraulic condition, rotational resistance, and rock-breaking response, respectively.

To further clarify the engineering relevance of the selected input variables, each parameter was interpreted according to the correlation analysis, permutation feature importance results, and drilling mechanics. Hole depth reflects the cumulative measured-depth effect, drill-string contact length, accumulated frictional resistance, and depth-related formation variations, which can affect WOB transmission and actual DWOB response. SWOB represents the surface-applied axial load and is the primary source of DWOB, although it is attenuated by drill-string–borehole-wall friction during transmission. Hook load reflects the overall drill-string loading state, including drill-string weight, buoyancy effect in drilling fluid, drag, and load-transfer efficiency. SPP reflects the hydraulic response and borehole circulation condition, which may indirectly affect DWOB through pressure fluctuation, hole-cleaning condition, and drilling-condition changes. The friction coefficient directly characterizes the frictional interaction between the drill string and the borehole wall and is a key factor controlling SWOB attenuation. Torque reflects drill-string rotational resistance, bit–rock interaction, borehole-wall contact, and local resistance in the horizontal section. ROP represents the rock-breaking response of the bit and provides feedback on effective downhole loading and formation response. Inclination angle describes wellbore trajectory and drill-string contact geometry; even slight variations in a horizontal section may affect normal contact force, frictional resistance distribution, and WOB transmission efficiency. Therefore, these variables were selected not only based on statistical correlation and feature importance, but also according to their physical relevance to drill-string load transfer and DWOB attenuation.

Although rotary speed was included in the candidate variables, its importance score was relatively low in the studied well section. In addition, its influence on WOB transmission can be partly reflected through torque and friction-related variables. Therefore, rotary speed was not retained in the final input set. By contrast, although the statistical importance of inclination angle was also relatively low, it has clear mechanical significance for describing wellbore trajectory, drill-string contact state, and frictional attenuation. Although the studied interval is a horizontal section and the inclination angle varies within a relatively narrow range, it was not treated as a constant in this study. If detailed inclination data are not available, the horizontal section may be approximately represented by a constant inclination angle in simplified engineering analysis. However, in this study, the measured inclination angle was available from the field data, and its slight variation still reflects local changes in wellbore trajectory and drill-string contact geometry. In horizontal wells, even small variations in inclination may affect the normal contact force between the drill string and borehole wall, the distribution of frictional resistance, and the efficiency of WOB transmission. Therefore, inclination angle was retained not as a dominant statistical predictor, but as a trajectory-related physical variable for describing drill-string contact state and frictional attenuation.

Considering statistical correlation, feature importance, and drill-string mechanical mechanisms, eight parameters were finally selected as model input variables: hole depth, SWOB, hook load, SPP, friction coefficient, torque, ROP, and inclination angle. The model output variable was DWOB. The feature correlation analysis and permutation feature importance analysis results are shown in Figure 4 and Figure 5, respectively, and the statistical ranges of the selected input parameters are listed in Table 1.

### 2.5. CNN-BiGRU Prediction Network

DWOB is affected by multiple drilling parameters acquired from surface sensors. Its variation depends not only on the local coupling relationships among drilling parameters at the current measured-depth point, but also on the drill-string loading state and accumulated frictional effects in adjacent well sections. Since the field drilling data used in this study were arranged sequentially along the measured-depth direction, the prediction task can be regarded as a depth-series modeling problem. Therefore, a CNN-BiGRU network was adopted as the basic prediction architecture to extract both local feature patterns and bidirectional depth-series dependencies from sensor parameter sequences.

Let the input sample obtained after preprocessing and sliding-window reconstruction be denoted as:

(3) $$X_{t}=R^{T\times d}$$

where *T* represents the window length of the depth-series sample and *d* is the number of input features. In this study, eight drilling parameters were selected as model inputs, including hole depth, SWOB, hook load, SPP, friction coefficient, torque, ROP, and inclination angle. The model output is the predicted DWOB.

First, a one-dimensional CNN was used to extract local features from the input sequence [30]. Unlike two-dimensional convolution, the one-dimensional convolution kernel slides along the measured-depth direction and captures local coupling relationships among multivariate drilling parameters within a short depth window through weighted aggregation. The output of the k-th convolution kernel at position *i* can be expressed as:

(4) $$c_{i}^{\left(k\right)}=\sigma \left(\sum_{j=0}^{m-1}W_{j}^{\left(k\right)}X_{i+j}+b^{\left(k\right)}\right)$$

where $c_{i}^{\left(k\right)}$ denotes the local feature extracted by the k-th convolution kernel at position i, m is the kernel size, $W_{j}^{\left(k\right)}$ and $b^{\left(k\right)}$ are the convolution kernel weight and bias, respectively, and $\sigma \left(\cdot \right)$ is the nonlinear activation function. After convolution and activation, a pooling layer was introduced to compress the extracted local features, reduce feature dimensionality, and improve the robustness of the model against local disturbances in sensor signals.

Although CNN can effectively extract local variation patterns from sensor parameter sequences, DWOB is also affected by the load transmission state and frictional accumulation in adjacent well sections. Therefore, a BiGRU was introduced after the CNN module to model the sequential dependency of the extracted feature sequence along the measured-depth direction. GRU uses an update gate and a reset gate to control the retention and forgetting of sequence information, thereby alleviating the gradient vanishing problem commonly encountered in traditional recurrent neural networks (RNN) during long-sequence modeling. The basic GRU calculations are given as follows:

(5) $$z_{t}=\sigma \left(W_{z}x_{t}+U_{z}h_{t-1}+b_{z}\right)$$

(6) $$r_{t}=\sigma \left(W_{r}x_{t}+U_{r}h_{t-1}+b_{z}\right)$$

(7) $${\tilde{h}}_{t}=tanh\left(W_{h}x_{t}+U_{h}\left(r_{t}⊙h_{t-1}\right)+b_{h}\right)$$

(8) $$h_{t}=\left(1-z_{t}\right)⊙h_{t-1}+z_{t}⊙{\tilde{h}}_{t}$$

where $z_{t}$ and $r_{t}$ denote the update gate and reset gate, respectively; $x_{t}$ is the input feature at time step *t*; $h_{t-1}$ is the hidden state at the previous time step; ${\tilde{h}}_{t}$ is the candidate hidden state; $h_{t}$ is the current hidden state; *W*, *U*, and *b* are the weight matrices and bias terms; and ⊙ denotes the Hadamard product.

BiGRU consists of a forward GRU and a backward GRU, enabling the model to extract sequence dependency information in both directions along the measured-depth sequence. For the input feature sequence, the forward GRU learns the state evolution from shallow to deep measured depth, whereas the backward GRU captures contextual information in the opposite direction within the same input window. The forward and backward hidden states can be expressed as:

(9) $${\vec{h}}_{t}=GRU\left(x_{t},{\vec{h}}_{t-1}\right)$$

(10) $${\overset{\leftarrow }{h}}_{t}=GRU\left(x_{t},{\overset{\leftarrow }{h}}_{t-1}\right)$$

(11) $$h_{t}=\left[{\vec{h}}_{t};{\overset{\leftarrow }{h}}_{t}\right]$$

where ${\vec{h}}_{t}$ and ${\overset{\leftarrow }{h}}_{t}$ are the forward and backward hidden states, respectively, and ([⋅;⋅]) denotes the concatenation operation.

It should be noted that the bidirectional structure of BiGRU is used only to enhance feature representation within each constructed depth-series input window, rather than to access future samples outside the current window. The forward GRU extracts sequential information along the increasing measured-depth direction, whereas the backward GRU encodes contextual information in the opposite direction within the same input window [31]. This bidirectional encoding does not use target values from future depth points, the validation set, or the test set, nor does it involve statistical information from the test data. In addition, the dataset was divided strictly according to the measured-depth order before model training, and no sliding window was allowed to cross the boundary between the training, validation, and test sets. The normalization parameters were calculated only from the training set and then applied to the validation and test sets. Therefore, the use of BiGRU improves the representation of local depth-series dependencies without causing data leakage during model training or evaluation.

Finally, the bidirectional features output by BiGRU were fed into a fully connected layer to map the high-dimensional feature representation to the predicted DWOB:

(12) $${\bar{y}}_{i}=W_{o}h_{t}+b_{o}$$

where ${\bar{y}}_{t}$ denotes the predicted DWOB, and $W_{o}$ and $b_{o}$ are the weight matrix and bias term of the output layer, respectively.

**Figure 6:**
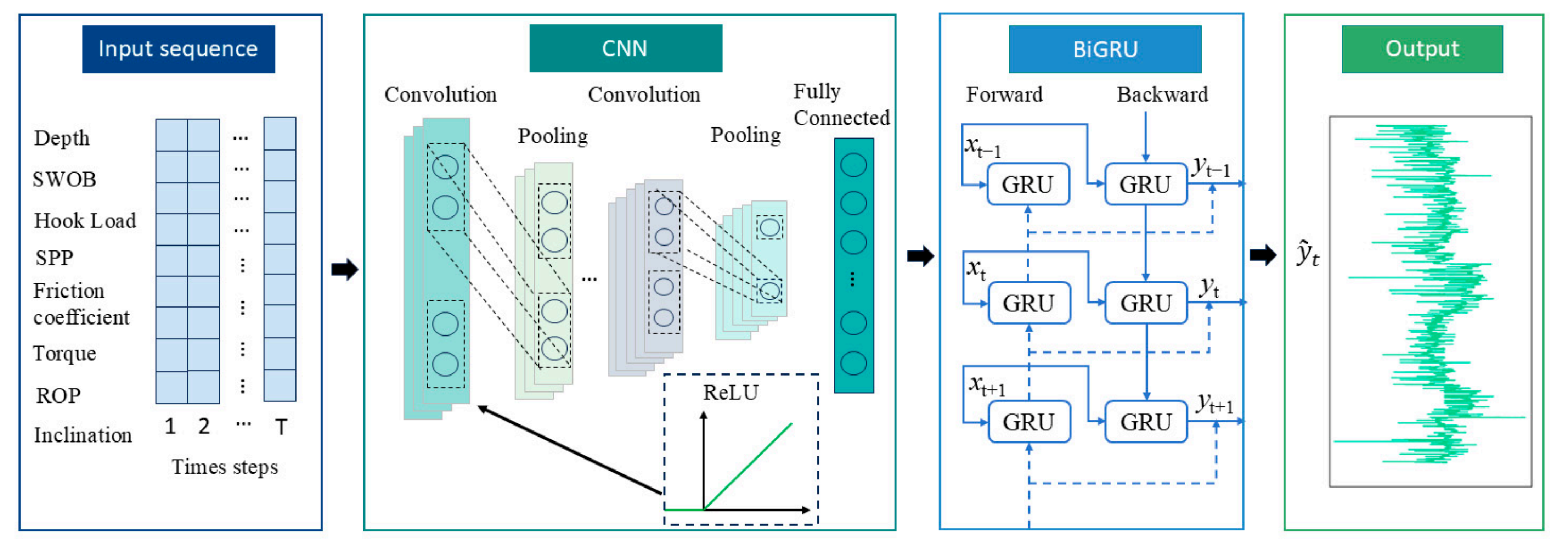
Schematic diagram of the CNN-BiGRU prediction network for DWOB prediction.

In summary, the CNN-BiGRU prediction network constructed in this study consists of an input sequence, a CNN-based local feature extraction module, a BiGRU-based bidirectional depth-series modeling module, and an output layer. The input sequence incorporates multiple parameters derived from surface sensors, such as hole depth, SWOB, hook load, SPP, torque, ROP and inclination angle. The friction coefficient, another input feature, is obtained via torque-and-drag calculation rather than direct sensor measurement. The CNN module is used to extract local coupling features between adjacent depth points, while the BiGRU module is employed to capture bidirectional dependencies of the drilling parameter sequence along the forward and backward measured-depth directions. Finally, the predicted DWOB is output through a fully connected layer. The structure of the CNN-BiGRU prediction network is shown in Figure 6.

### 2.6. Design of the Physics-Informed Loss Function

Although purely data-driven models have strong nonlinear fitting capability, their predictions depend mainly on the data distribution and lack explicit constraints from drill-string mechanical laws. When MWD data contain strong noise, abrupt changes, or non-stationary operating conditions, the model may generate nonphysical predictions in order to reduce the data-fitting error. Therefore, the simplified drill-string frictional attenuation relationship introduced in Section 2.2 is embedded into the loss function as a physical prior to improve the physical consistency of DWOB prediction.

For the *i*-th sample, the measured DWOB is denoted as $y_{i}$, and the neural network prediction is denoted as ${\tilde{y}}_{i}$. The data loss is defined as the mean squared error between the predicted and measured DWOB:

(13) $$L_{data}=\frac{1}{N}\sum_{i=1}^{N}{\left(y_{i}-{\tilde{y}}_{i}\right)}^{2}$$

where *N* is the number of samples, $y_{i}$ is the measured DWOB of the *i*-th sample, and ${\tilde{y}}_{i}$ is the corresponding predicted value.

To impose the drill-string mechanical constraint, the physics-based DWOB calculated from the simplified frictional attenuation prior is introduced as a reference value. For the *i*-th sample, it is expressed as:

(14) $$y_{i}^{phys}={SWOB}_{i}\cdot e^{\left(-\mu_{s,i},\eta_{b,i}\right)}$$

where $y_{i}^{phys}$ is the physics-based reference value of DWOB for the *i*-th sample, ${SWOB}_{i}$ is the SWOB, $\mu_{s,i}$ is the equivalent friction coefficient, and $\eta_{b,i}$ is the inclination-related equivalent contact attenuation coefficient. The equivalent friction coefficient was obtained through the torque-and-drag model-based calibration described in Section 2.4, rather than from direct MWD measurement.

The simplified attenuation relationship was adopted from the torque-and-drag-related WOB transmission model reported in previous studies [30]. In this study, the equation was used as a physics-based reference to describe the general attenuation trend from SWOB to DWOB in a horizontal well. The main assumptions are that the attenuation of WOB transmission is primarily governed by drill-string–borehole-wall contact, frictional resistance and wellbore inclination. The friction coefficient is treated as an equivalent friction coefficient calibrated from field hook load matching, and the attenuation relationship is assumed to represent the quasi-static load-transfer trend along the measured-depth interval. This simplified model does not explicitly describe transient drill-string vibration, bit bounce, stick-slip, complex cuttings-bed evolution, drilling-fluid rheology, or local formation heterogeneity. Therefore, it is not used to replace the measured DWOB data or a complete three-dimensional drill-string dynamics model; instead, it is embedded into the loss function as a physical constraint to guide the predicted DWOB toward a mechanically reasonable attenuation trend.

The physics loss is then defined as the mean squared error between the neural network prediction and the physics-based DWOB:

(15) $$L_{phys}=\frac{1}{N}{\sum_{i=1}^{N}({\tilde{y}}_{i}-y_{i}^{phys})}^{2}$$

The total loss function consists of the data loss and the physics loss:

(16) $$L_{total}=L_{data}+\gamma L_{phys}$$

where $L_{total}$ is the total loss, $L_{data}$ is the data-fitting loss, $L_{phys}$ is the physics-constrained loss, and γ is the physics-constrained weight coefficient used to balance the relative contributions of data fitting and physical consistency.

In this formulation, $L_{data}$ is calculated from the error between the model-predicted DWOB and the field-measured DWOB, whereas $L_{phys}$ is calculated from the error between the model-predicted DWOB and the physics-based DWOB reference value derived from the drill-string frictional attenuation relationship. The field-measured DWOB is used as the supervised learning label, while the physics-based DWOB reference value is used only in the physics-constrained loss term to provide a mechanical constraint during model training. Therefore, the training objective is not only to fit the field-measured DWOB data, but also to guide the predicted DWOB to follow the physics-based attenuation trend.

The physical constraint weight γ is used to adjust the contribution of the physics-informed loss term to the total loss function. In other words, γ controls the balance between the data-fitting loss and the physics-informed loss during model training. A smaller γ weakens the physical constraint, whereas a larger γ increases the influence of the drill-string frictional attenuation relationship. The model parameters are updated by minimizing the total loss function, so an appropriate γ is important for balancing prediction accuracy and physical consistency. In this study, γ was treated as a tunable hyperparameter and determined according to the validation-set performance during the optimization process. The search range of γ was set as [0.02, 2], and the final optimized value was 0.1.

**Figure 7:**
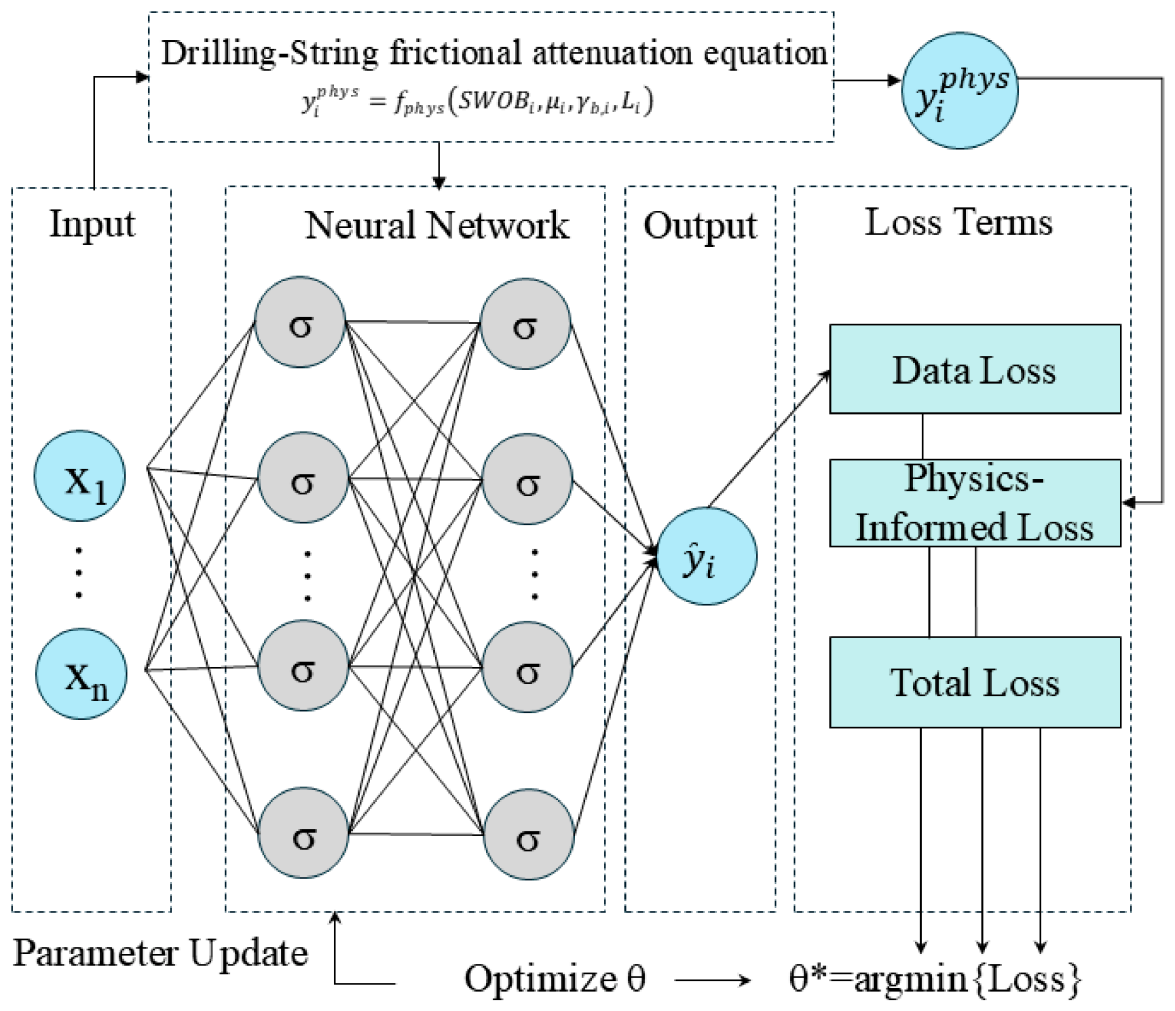
Schematic framework of the physics-informed loss function and parameter update process.

During each forward propagation process, the CNN-BiGRU network outputs the predicted DWOB according to the input drilling parameters. Meanwhile, the corresponding physics-based DWOB is calculated using the drill-string frictional attenuation equation. The predicted value is compared with both the measured value and the physics-based value to obtain the data loss and physics loss, respectively. These two loss terms are then combined to form the total loss, which is used for backpropagation and parameter updating. In the schematic diagram, the superscript * in $\theta^{*}$ denotes the optimal parameter set obtained by the whale optimization algorithm. The schematic framework of the physics-informed loss function is shown in Figure 7. Compared with a purely data-driven loss function, the proposed physics-informed loss function not only minimizes the prediction error but also constrains the model output to follow the basic mechanical behavior of drill-string frictional attenuation. This joint data–physics optimization mechanism reduces the influence of noisy measurements and decreases the possibility of nonphysical predictions under complex drilling conditions.

### 2.7. WOA-Based Adaptive Hyperparameter Optimization

In PINNs, both network structural parameters and the physics-constrained weight have significant effects on model prediction performance. If the hidden dimension is too small, the model may be unable to sufficiently learn complex nonlinear features, resulting in underfitting. Conversely, an excessively large hidden dimension may introduce redundant parameters and increase the risk of overfitting. The learning rate determines the update step of network parameters; a learning rate that is too large may cause training oscillation, whereas a learning rate that is too small may reduce convergence efficiency. In addition, the physics-constrained weight γ directly controls the balance between the data loss and the physics loss. An inappropriate value of γ may cause the model to rely excessively on data fitting or over-conform to the simplified physical equation, thereby affecting the overall prediction performance.

To reduce the uncertainty caused by empirical parameter tuning, the WOA is introduced in this study to perform global optimization of key model hyperparameters [32]. WOA is a swarm intelligence optimization algorithm inspired by the hunting behavior of humpback whales. It has the advantages of few control parameters, simple implementation, and strong global search capability. During the iterative optimization process, WOA dynamically balances global exploration and local exploitation through encircling prey, spiral updating, and random search mechanisms, thereby improving its ability to identify the optimal parameter combination.

In this study, the key hyperparameters affecting model performance are mapped to the position vector of each whale individual, which can be expressed as:

(17) $$X=\left[H,\eta ,K,\gamma ,\lambda \right]$$

where *H* denotes the hidden dimension of BiGRU, η is the learning rate, *K* is the number of convolution kernels, γ is the physics-constrained weight, and λ is the regularization coefficient.

The composite loss on the validation set is used as the fitness function to guide the WOA search process:

(18) $$F\left(X\right)=L_{val}=L_{data}^{val}+\gamma L_{phys}^{val}$$

During the encircling phase, the distance between the current whale individual and the best individual is calculated as:

(19) $$D=\left|C\cdot X^{*}(t)-X(t)\right|$$

and the position of the whale individual is updated according to:

(20) $$X\left(t+1\right)=X^{*}(t)-A\cdot D$$

where X(t) is the current position vector, $X^{*}(t)$ represents the current best position vector, *A* and *C* are coefficient vectors defined as:

(21) A=2ar−a,C=2r

where *a* decreases linearly during iterations and γ is a random vector within [0, 1]. Through iterative position updating, WOA continuously searches for the hyperparameter combination that minimizes the validation loss. When the fitness value reaches the minimum or the maximum number of iterations is satisfied, the optimal hyperparameter configuration is obtained and used for subsequent model training and testing.

By introducing WOA, the proposed model can adaptively determine both the structural parameters of the deep network and the physics-constrained weight. This reduces the subjectivity of manual parameter tuning and helps alleviate the imbalance between the data loss and the physics loss, thereby improving model convergence stability and prediction accuracy.

**Figure 8:**
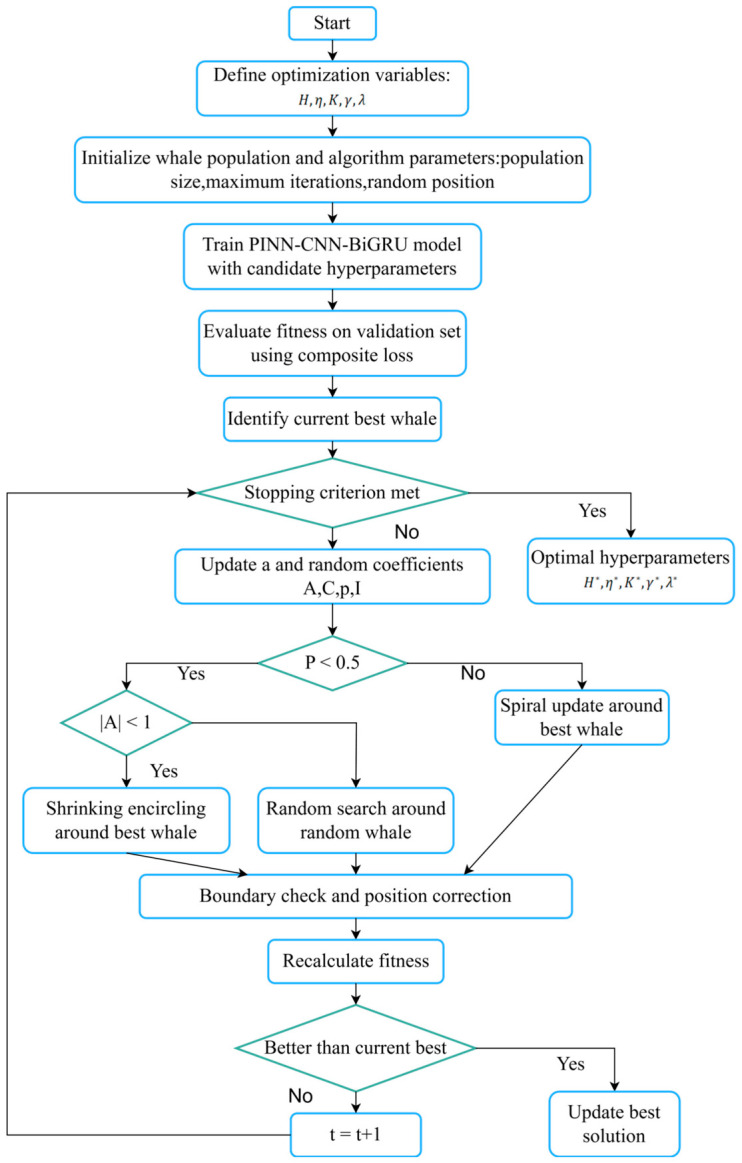
WOA-based hyperparameter and physics-constrained weight optimization procedure for the proposed PINN-CNN-BiGRU model.

To further clarify the coupling relationship between WOA and the proposed physics-informed CNN-BiGRU model, the WOA-based optimization procedure is illustrated in Figure 8. In the figure, the superscript * on the parameters (e.g., ($H^{*}$, $\eta^{*}$, $K^{*}$, $\gamma^{*}$, $\lambda^{*}$)) denotes their optimal values identified by the whale optimization algorithm. In this procedure, each whale individual represents a candidate hyperparameter vector, including the BiGRU hidden dimension, learning rate, number of CNN convolution kernels, physics-constrained weight, and L2 regularization coefficient. For each candidate solution, the PINN-CNN-BiGRU model is trained on the training set, and the composite loss on the validation set is used as the fitness value. Through iterative position updating, WOA searches for the hyperparameter combination that minimizes the validation loss. The optimal hyperparameter configuration is then used for final model training and testing.

All data processing, feature analysis, model construction, training, performance evaluation and graphical visualization in this study were implemented using Python (Version 3.11.14), pandas (Version 2.3.3), NumPy (Version 2.3.4), PyTorch (Version 2.11.0+cpu), scikit-learn (Version 1.8.0) and Matplotlib (Version 3.10.6).

## 3. Model Validation and Analysis

### 3.1. Dataset Description

The proposed model was trained, validated, and tested using field surface sensor data from an actual horizontal well. The dataset was collected sequentially along the measured-depth direction during drilling operations. After data preprocessing, including outlier removal, missing-value interpolation, filtering and denoising, normalization, and sliding-window reconstruction, a total of 9957 valid samples were obtained. The measured-depth interval of the processed dataset ranged from approximately 2506.43 m to 3750 m, with an average sampling interval of about 0.1 m along the depth direction.

The input variables included hole depth, SWOB, hook load, SPP, friction coefficient, torque, ROP, and inclination angle. These variables were obtained from surface sensor measurements and an equivalent engineering parameter. The target output was the field-measured DWOB. Since the drilling data were continuously acquired along the measured-depth direction and exhibited strong sequential correlation, the dataset was divided strictly according to the measured-depth order rather than by random sampling. Specifically, approximately 70% of the samples were used for model training, 10% were used for validation, and the remaining 20% were reserved for final testing.

The validation set was used for WOA fitness evaluation, learning-rate adjustment, early stopping, and model selection, whereas the test set was used only for final model evaluation. This splitting strategy avoids data leakage caused by random partitioning and is more consistent with the engineering scenario in which data from drilled sections are used to predict DWOB variations in subsequent well sections. To further prevent information leakage, the normalization parameters, including the minimum and maximum values of each input variable, were calculated only from the training set. The same normalization parameters were then applied to the validation and test sets.

### 3.2. Model Training Configuration

During the model training process, the WOA was first employed to perform global optimization of the key hyperparameters. Once the optimal hyperparameter combination was obtained, it was incorporated into the WOA–PINN–CNN–BiGRU model for formal training. The network was trained using the Adam optimizer, combined with L_2_ regularization, adaptive learning rate decay, and an early stopping mechanism to enhance training stability and prevent overfitting.

When the validation loss ceased to decrease over a consecutive number of training epochs, the early stopping mechanism was triggered, and the model parameters corresponding to the minimum validation loss were retained as the final model. This strategy prevents the model from overfitting on the training set while improving its generalization capability on the test set.

### 3.3. Comparative Models and Ablation Experiment Setup

To evaluate the effectiveness of the proposed sensor-data-driven prediction framework, the WOA-PINN-CNN-BiGRU model was compared with several representative benchmark models, including traditional machine learning models, namely random forest (RF) and support vector machine (SVM), as well as commonly used deep learning sequence models, including TCN, RNN, LSTM, and GRU.

To ensure a fair comparison, all models were trained, validated, and tested using the same preprocessed sensor dataset, the same input variables, and the same depth-ordered data partitioning strategy. Specifically, 6970 samples were used for training, 996 samples were used for validation, and 1991 samples were used for testing. The validation set was used for hyperparameter selection, WOA fitness evaluation, learning-rate adjustment, and early stopping, whereas the test set was used only for final performance evaluation. The normalization parameters were calculated only from the training set and then applied to the validation and test sets, ensuring that no statistical information from the validation or test sets was involved in model training.

For all benchmark models, the same evaluation metrics, including MRE, MAE, RMSE, and R^2^, were used to assess the prediction performance on the same test set. The main hyperparameters of the baseline models were determined based on the validation performance under the same evaluation framework. Therefore, the comparison focuses on the influence of different sensor-data-driven modeling strategies on DWOB prediction under consistent data preprocessing, feature input, dataset partitioning, and evaluation conditions.

Furthermore, to analyze the contribution of each core component in the proposed framework, a progressive ablation experiment was designed. The ablation models included CNN-GRU, CNN-BiGRU, PINN-CNN-GRU, PINN-CNN-BiGRU, WOA-PINN-CNN-GRU, and the complete WOA-PINN-CNN-BiGRU model. By gradually introducing the BiGRU structure, the physics-informed constraint, and the WOA-based optimization strategy, the influence of each component on DWOB prediction accuracy and error distribution was examined under a controlled comparison framework.

### 3.4. Evaluation Metrics

To comprehensively evaluate the prediction performance of the model, four evaluation metrics were selected: MRE, MAE, RMSE, and R^2^. Specifically, MRE measures the average relative deviation of the predicted values from the actual values; MAE reflects the average magnitude of the prediction errors; RMSE is more sensitive to large errors and can indicate the model’s ability to control anomalous deviations; and R^2^ evaluates the overall goodness of fit between the predicted and actual values. In general, lower values of MRE, MAE, and RMSE, together with an R^2^ closer to 1, indicate superior prediction performance. The formulas for each evaluation metric are presented in Equations (22)–(25).

(22) $$MRE=\frac{1}{n}\sum_{i=1}^{n}\left|\frac{y_{i}-{\tilde{y}}_{i}}{y_{i}}\right|$$

(23) $$MAE=\frac{1}{n}\sum_{i=1}^{n}\left|{y_{i}-\tilde{y}}_{i}\right|$$

(24) $$RMSE=\sqrt{\frac{1}{n}\sum_{i=1}^{n}{\left({y_{i}-\tilde{y}}_{i}\right)}^{2}}$$

(25) $$R^{2}=\frac{{\left[\sum_{i=1}^{n}\left(y_{i}-\bar{y}\right)\left({\tilde{y}}_{i}-\bar{\tilde{y}}\right)\right]}^{2}}{\sum_{i=1}^{n}{\left(y_{i}-\bar{y}\right)}^{2}\sum_{i=1}^{n}{\left({\tilde{y}}_{i}-\bar{\tilde{y}}\right)}^{2}}$$

## 4. Results and Discussion

### 4.1. WOA Hyperparameter Optimization Results and Training Convergence Analysis

The eight selected drilling parameters were fed into the WOA–PINN–CNN–BiGRU model, and the WOA was employed to perform global optimization of the model’s key hyperparameters. The WOA configuration was set as follows: a population size of 30, a maximum of 150 iterations, a hidden layer dimension search range of [16, 256], and a learning rate search range of [0.0001, 0.01]. The composite loss function on the validation set was used as the fitness function to drive the WOA in searching for the optimal hyperparameter combination.

The WOA optimization results indicate that the model achieved a favorable parameter configuration within the given search space. The optimal unidirectional hidden layer dimension of the BiGRU was 36, corresponding to a fully connected layer input dimension of 72; the number of CNN convolution kernels was 19 with a kernel size of 3; the number of stacked BiGRU layers was three; the physics-constrained weight coefficient γ was 0.1; the learning rate was 3.17 × 10^−3^; the L2 regularization coefficient was 1 × 10^−6^; and the optimal fitness value was 3.371 × 10^−2^. The optimal hyperparameter configuration is summarized in Table 2.

**Figure 9:**
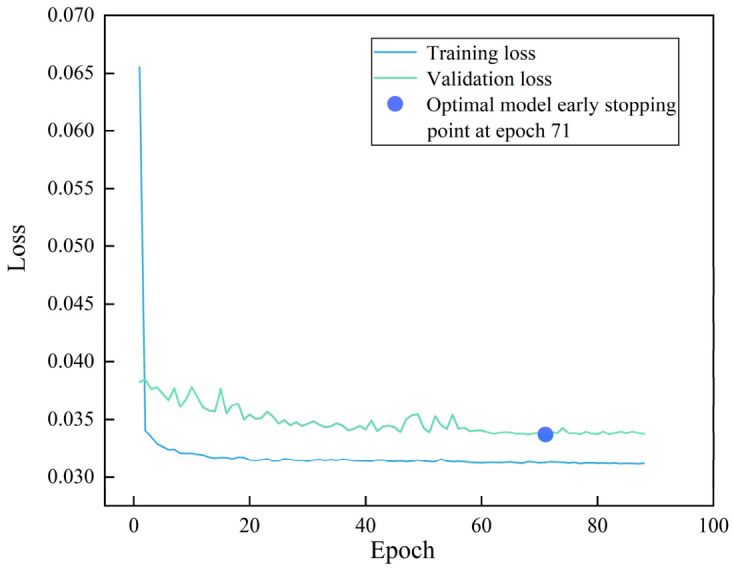
Training and validation loss convergence curves of the proposed model.

Once the optimal hyperparameters were determined, they were incorporated into the model for training. During the training process, the Adam optimizer, a learning rate plateau decay strategy, and an early stopping mechanism were adopted. The training and validation loss curves are presented in Figure 9. As shown in the figure, both the training loss and the validation loss exhibited a decreasing trend with the increasing number of training epochs, indicating that the model was able to effectively learn the nonlinear mapping relationship between the drilling parameters and the DWOB. The model reached the minimum composite loss on the validation set at the 71st epoch; subsequently, the validation loss did not decrease further over 20 consecutive epochs, and early stopping was triggered at the 91st epoch. Ultimately, the model parameters corresponding to the minimum validation loss were retained for prediction on the test set.

### 4.2. Performance Comparison of Different Prediction Models

**Figure 10:**
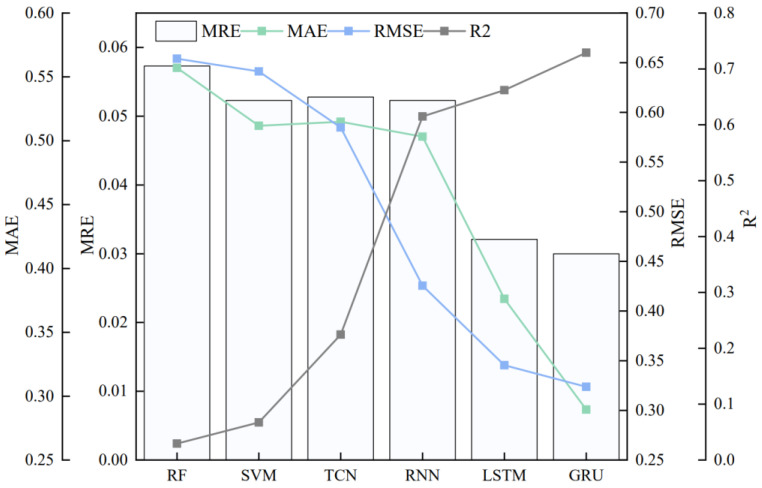
Performance comparison of different DWOB prediction models.

To evaluate the predictive performance of the proposed sensor-data-driven model under consistent data conditions, the WOA-PINN-CNN-BiGRU model was compared with several benchmark models, including RF, SVM, TCN, RNN, LSTM, and GRU. All models used the same sensor input variables, preprocessing procedure, depth-ordered training-validation-test split, and evaluation metrics. The prediction performance of each model on the test set is shown in Figure 10.

As shown in Figure 10, the traditional machine learning models, including RF and SVM, show relatively lower prediction accuracy than the sequence-based deep learning models. This indicates that models without sequence modeling capability may have difficulty capturing the depth-dependent variation characteristics of DWOB from continuous sensor parameter sequences. TCN achieved an MRE of 0.0528, MAE of 0.5149, RMSE of 0.5848, and $R^{2}$ of 0.2244. Compared with RF and SVM, TCN shows improved RMSE and $R^{2}$, indicating that temporal convolution can capture certain local depth-series dependencies. However, its overall performance is still lower than that of the recurrent neural-network models. This may be because the DWOB response in horizontal wells is affected by cumulative load transfer and frictional attenuation along the measured-depth direction, for which recurrent structures are more effective under the present field-data conditions. In comparison, RNN, LSTM, and GRU obtain improved prediction results, suggesting that sequence modeling is beneficial for representing the relationship between sensor-measured drilling parameter variations and DWOB along the measured-depth direction.

Among the benchmark models, GRU shows relatively better overall performance than RNN and LSTM on the present dataset. This may be attributed to its gating mechanism, which helps retain useful sequence information while maintaining a relatively simple network structure. Compared with the benchmark models, the proposed WOA-PINN-CNN-BiGRU model achieves the best performance among the tested models, with an R^2^ of 0.9245 and RMSE, MAE, and MRE values of 0.1856, 0.1135, and 0.0124, respectively. These results indicate that the combination of CNN-BiGRU-based sensor sequence feature extraction, physics-informed constraints, and WOA-based parameter optimization can improve the prediction performance of DWOB under the tested field-data conditions.

It should be noted that the comparison is based on a single horizontal well dataset. Therefore, the results mainly demonstrate the effectiveness of the proposed modeling framework for the present MWD sensor dataset, while further validation using multi-well datasets is still needed to evaluate its broader generalization capability under more diverse drilling conditions.

### 4.3. Analysis of Ablation Experiments

**Figure 11:**
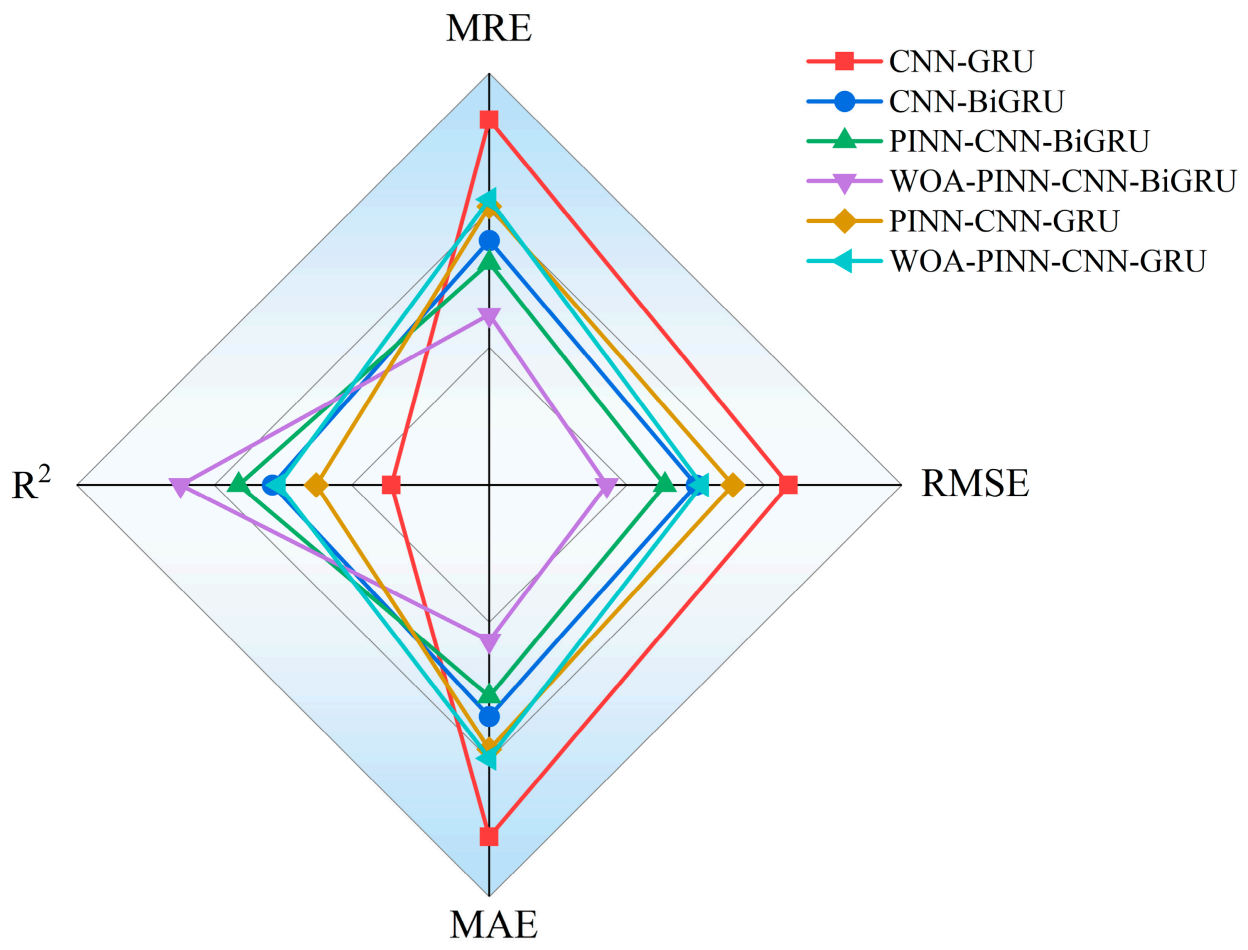
Comparison of ablation experiment prediction performance for different model variants.

To further examine the contribution of different components in the proposed framework, ablation experiments were conducted by gradually introducing the BiGRU structure, the physics-informed constraint, and the WOA-based optimization strategy. The prediction performance of different model variants is shown in Figure 11.

As shown in Figure 11, the CNN–BiGRU model achieves better prediction performance than the CNN–GRU model on the present dataset, with R^2^ increasing from 0.7711 to 0.8577 and MRE decreasing by 33.1%. This result suggests that the BiGRU structure can enhance the representation of depth-dependent sequence features and improve the model’s ability to capture the variation characteristics of DWOB along the measured-depth direction. However, this improvement should be interpreted within the scope of the current dataset and model settings.

After introducing the physics-informed constraint, the prediction performance of the PINN–CNN–BiGRU model is further improved, with R^2^ increasing to 0.8823 and both RMSE and MAE decreasing. This indicates that embedding the drill-string frictional attenuation relationship into the loss function as a physical prior can provide an additional mechanical constraint for the neural network output. As a result, the model tends to produce predictions that are more consistent with the basic mechanical behavior of WOB transmission, rather than relying solely on data fitting.

After WOA-based hyperparameter optimization is incorporated, the WOA–PINN–CNN–BiGRU model achieves the best overall performance among the tested model variants. Compared with the PINN–CNN–BiGRU model without WOA optimization, the complete model shows further improvements in R^2^, RMSE, MAE, and MRE. This suggests that WOA helps obtain a more suitable combination of network hyperparameters and physics-constrained weight for the present prediction task, thereby improving the coordination between data-fitting accuracy and physical consistency.

Overall, the ablation results indicate that each component contributes to the final prediction performance to different extents. The CNN-BiGRU structure improves the extraction of local and sequence features from depth-series drilling data, the physics-informed constraint enhances the mechanical consistency of the prediction results, and WOA optimization improves the parameter configuration of the hybrid model. Nevertheless, these conclusions are based on the current single-well dataset, and further validation with additional wells is required to assess the robustness and generalization capability of each module under more diverse drilling conditions.

### 4.4. Sensitivity Analysis of the Physical Constraint Weight

To further evaluate the influence of the physical constraint weight γ, a sensitivity analysis was conducted within the WOA search range [0.02, 2]. In this analysis, the optimized network hyperparameters were kept unchanged, and only γ was varied. The tested values included γ=0.02, γ=0.05, γ=0.1, γ=0.2, and γ=0.5, representing the lower-bound weak-constraint case, the optimized value, and stronger physical-constraint cases, respectively. The purpose of this analysis was to examine the effect of γ on model performance rather than to re-evaluate the effect of WOA, which has been discussed in the ablation experiments. The prediction performance was evaluated using MRE, RMSE, MAE, and R^2^, as shown in Table 3.

As shown in Table 3, the prediction performance varies with the physical constraint weight γ. When γ = 0.02, the physics-constrained loss term exerts only a weak influence on model training, and the prediction mainly relies on the data-fitting loss. As γ increases, the drill-string frictional attenuation relationship gradually contributes to model training. The results show that γ = 0.1 provides the best or near-best prediction performance among the tested values, indicating that an appropriate physical constraint weight can improve the balance between data fitting and physical consistency. However, when γ becomes too large, the model may be over-constrained by the simplified attenuation relationship, which can reduce its ability to fit complex nonlinear field data.

### 4.5. Regression Analysis of Predicted and Actual Values

**Figure 12:**
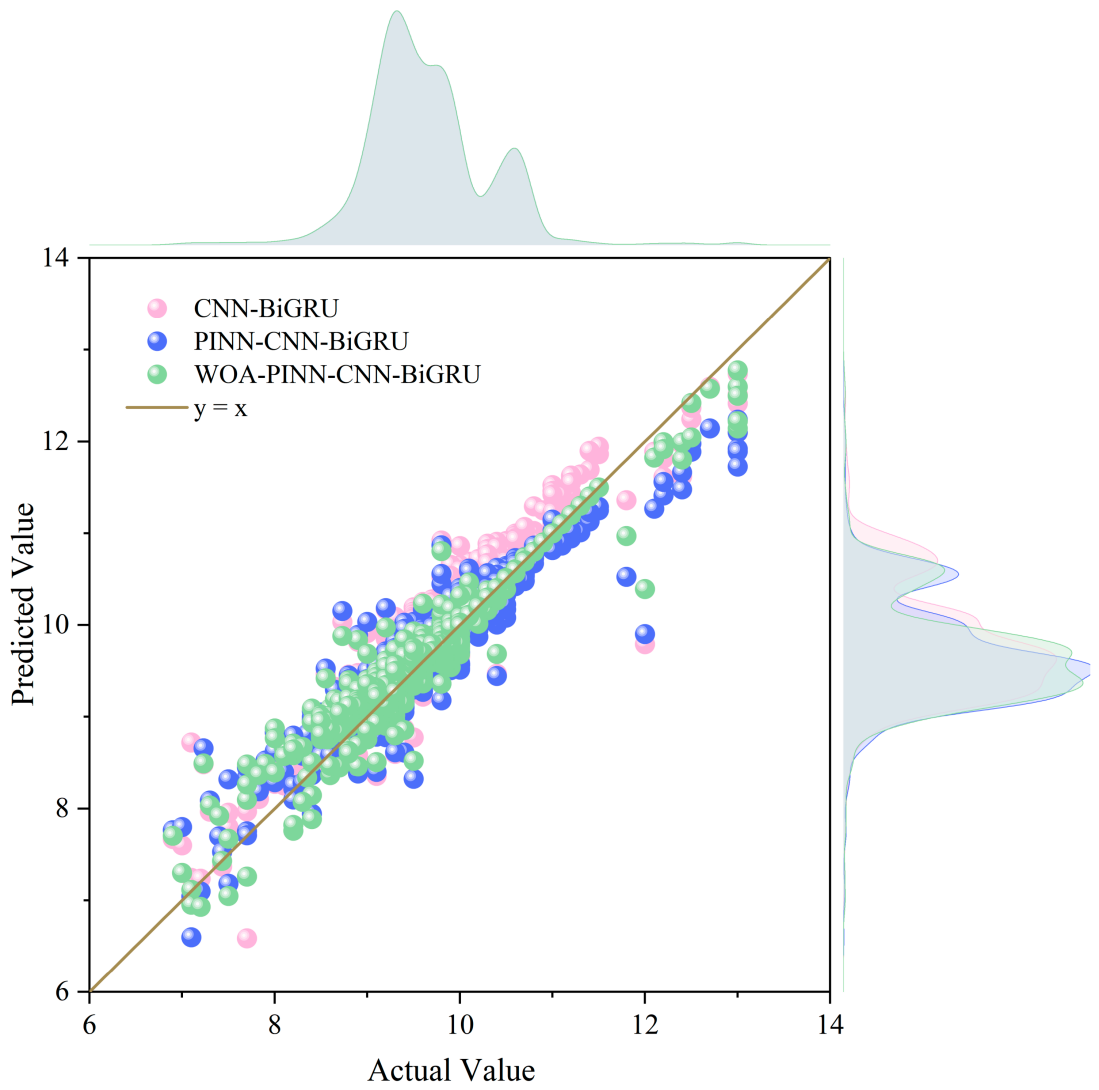
Comparison of regression scatter plots and marginal distributions between predicted and measured values for representative models.

To further analyze the consistency between the predicted values and the measured DWOB, regression scatter plots with marginal distributions were drawn for representative models, as shown in Figure 12. The shaded areas above and to the right of the scatter plot are kernel density distributions, which illustrate the probability distributions of the measured values (horizontal axis) and predicted values (vertical axis) for each model, respectively. The figure compares the prediction results of three models, namely CNN-BiGRU, PINN-CNN-BiGRU, and WOA-PINN-CNN-BiGRU, with the ideal line y = x used as a reference.

As shown in Figure 12, the scatter points of the purely data-driven CNN-BiGRU model are relatively dispersed, and some samples deviate from the ideal diagonal line, indicating that certain discrepancies still exist between the predicted and measured values. After introducing physics-informed constraints, the scatter points of the PINN-CNN-BiGRU model become more concentrated around the ideal diagonal line, suggesting that the physical prior can effectively constrain nonphysical deviations in the model outputs. The complete WOA-PINN-CNN-BiGRU model shows the most concentrated scatter distribution and the highest consistency between the predicted and measured values, demonstrating stronger fitting capability and prediction stability.

In addition, the marginal error distributions indicate that the prediction errors of the complete model are more concentrated, with fewer abnormal scattered points. This further demonstrates that the combination of WOA optimization and physics-informed constraints can enhance the model’s ability to suppress abnormal prediction errors.

### 4.6. Dynamic Prediction Performance Under Unsteady Working Conditions

**Figure 13:**
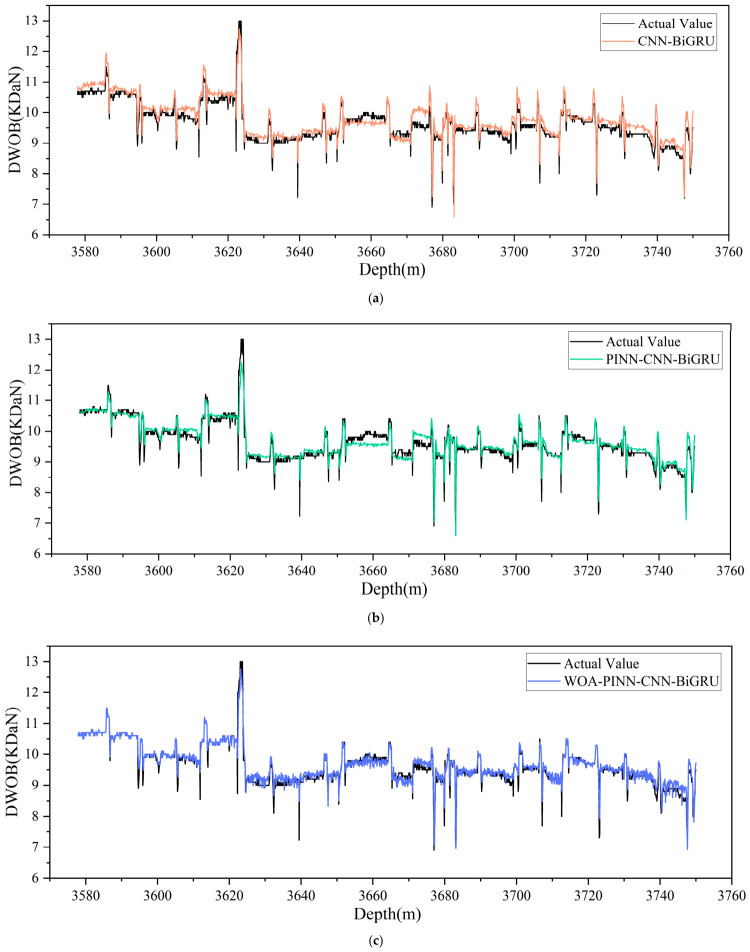
Dynamic prediction comparison of DWOB in the test well section using different models: (**a**) CNN-BiGRU; (**b**) PINN-CNN-BiGRU; (**c**) WOA-PINN-CNN-BiGRU.

To evaluate the dynamic response capability of the model under actual non-stationary drilling conditions, the DWOB prediction results in the test well section were further selected for depth-series comparison, as shown in Figure 13. Figure 13 presents the comparison results between the measured DWOB and the predicted values obtained by three representative models, namely CNN-BiGRU, PINN-CNN-BiGRU, and WOA-PINN-CNN-BiGRU.

As shown in Figure 13, in well sections where the DWOB varies relatively smoothly, all three models can generally track the overall trend of the measured curve. However, at positions with abrupt WOB changes, local peaks, and valleys, the purely data-driven CNN-BiGRU model exhibits certain prediction lag and local overshoot, indicating its limited adaptability to complex non-stationary operating conditions. After introducing physics-informed constraints, the oscillations of the PINN-CNN-BiGRU model near abrupt-change points are reduced, and the agreement between the predicted curve and the measured curve is improved. The complete WOA-PINN-CNN-BiGRU model shows better performance in tracking peaks, valleys, and the overall trend, and its prediction results are the most consistent with the variation pattern of the field-measured DWOB.

In this study, prediction lag and local overshoot were identified by visually comparing the predicted DWOB curves with the measured DWOB curve along the measured-depth direction. Prediction lag refers to the delayed response of the predicted curve relative to the measured curve near abrupt increases, decreases, or local extrema of DWOB. Local overshoot refers to the phenomenon where the predicted curve exceeds the corresponding measured peak or valley in a local fluctuation interval. Therefore, the comparison focused on whether the predicted curve could follow the position, direction, and amplitude of local DWOB variations.

These observations indicate that the physics-informed constraints help the model outputs better conform to the attenuation law of WOB transmission, while WOA optimization further improves the model parameter configuration. Therefore, the proposed method can improve prediction stability in the tested non-stationary intervals. Further validation using multi-well datasets is still required to evaluate its robustness under more diverse drilling conditions.

### 4.7. Discussion on Engineering Applicability and Limitations

From the perspective of sensor-based drilling monitoring, the proposed WOA-PINN-CNN-BiGRU model can estimate DWOB using surface sensor data and calibrated drilling engineering parameters, providing a feasible data–physics hybrid solution when continuous direct DWOB measurements are limited or difficult to obtain. Compared with traditional mechanical models, the developed model shows stronger nonlinear mapping capability and better adaptability to field data under the tested conditions. Compared with purely data-driven models, the introduction of the drill-string frictional attenuation relationship improves the physical consistency and interpretability of the prediction results. Therefore, the proposed method has the potential to provide auxiliary decision support for WOB adjustment, drilling parameter optimization, and future intelligent drilling control in horizontal wells.

Nevertheless, several limitations remain in the present study. First, the model validation was conducted using field data from a single horizontal well, and the coverage of data sources and operating conditions is still limited. In future work, the generalization capability of the model should be further evaluated using datasets from different well blocks, well types, and drilling conditions. Second, the present model does not explicitly incorporate drilling-fluid properties, annular flow-regime information, cuttings transport, or hole-cleaning conditions, because continuous and depth-synchronized records of these factors were not available in the field dataset. Although SPP, torque, hook load, ROP, and the equivalent friction coefficient may partially and indirectly reflect some hydraulic response, borehole-condition, and frictional effects, they cannot replace direct measurements of drilling-fluid properties, flow-regime characteristics, or cuttings-transport conditions. Therefore, the lack of explicit drilling-fluid and cuttings-transport parameters remains a limitation of the present model. Future work will consider incorporating these factors when continuous and synchronized field measurements are available. Third, the present study focuses on DWOB prediction under normal rotary drilling conditions and does not explicitly model abnormal drilling events such as drill pipe sticking. The field dataset used in this study did not contain clearly labeled stuck-pipe events or abnormal-condition annotations. Therefore, the proposed model should be regarded as a DWOB prediction model for normal rotary drilling conditions rather than a stuck-pipe diagnosis or early-warning model. In subsequent work, abnormal-condition data will be incorporated to further integrate DWOB prediction with drilling-safety warning and diagnosis.

## 5. Conclusions

Aiming to address the problems of large drill-string frictional resistance, severe WOB transmission loss, and insufficient physical consistency of purely data-driven prediction models during extended-reach horizontal well drilling, this study proposed a WOA-PINN-BiGRU model for DWOB prediction using surface sensor data and calibrated drilling engineering parameters. The drill-string frictional attenuation relationship was embedded into the neural network loss function as a physical prior, and WOA was used to optimize the network hyperparameters and the physics-constrained weight. The proposed model integrates sensor-data-driven feature learning with drilling mechanical constraints. The main conclusions are as follows:

1. A PINN incorporating the drill-string frictional attenuation relationship was established for DWOB prediction. By embedding the physical constraint term into the loss function, the model can fit field-measured drilling data while maintaining consistency with the basic mechanical behavior of WOB transmission. The field-data validation results indicate that the physical constraint can help restrict the solution space of the neural network and improve the physical consistency of the prediction results.
2. The CNN-BiGRU structure improved the prediction performance of DWOB on the present dataset. The CNN module extracts local feature information from sensor parameter sequences, while the BiGRU module captures bidirectional depth-series dependencies associated with DWOB variation. According to the ablation experiment results, compared with the CNN-GRU model, the CNN-BiGRU model increased the coefficient of determination R^2^ from 0.7711 to 0.8577 and reduced MRE by 33.1%. This result suggests that bidirectional sequence modeling is beneficial for representing the depth-dependent variation characteristics of DWOB.
3. WOA-based optimization further improved the performance of the physics-informed model under the tested conditions. By optimizing the network hyperparameters and the physics-constrained weight, the complete WOA-PINN-CNN-BiGRU model achieved the best performance among the tested model variants. Its R^2^ reached 0.9245, while RMSE, MAE, and MRE were 0.1856, 0.1135, and 0.0124, respectively. These results indicate that the combination of physics-informed constraints and WOA-based parameter optimization can improve the coordination between data fitting and physical consistency.
4. The proposed model showed good dynamic tracking capability in the test well section. The depth-series prediction results indicate that, compared with purely data-driven models, the proposed model can reduce prediction lag and local overshoot to some extent in intervals with abrupt WOB fluctuations. The predicted curve is more consistent with the variation trend of the measured DWOB. Therefore, the proposed method provides a potential technical basis for sensor-based DWOB prediction and drilling parameter optimization in horizontal well drilling.

## Figures and Tables

**Table 1:** Statistical ranges of the selected input features.

Feature	Unit	Range
Hole depth	m	2506.43–3750
SWOB	kDaN	8.0–16.0
Hook Load	kDaN	43.4–52.8
SPP	kPa	23,519–31,404
Friction Coefficient	-	0.09–0.2
Torque	N⋅m	6561.93–13,740.77
ROP	m/h	14.46–65.93
Inclination	rad	1.46–1.59

**Table 2:** Optimal hyperparameter configuration of the WOA–PINN–CNN–BiGRU model.

Module	Hyperparameter	Optimized Value
CNN	Number of convolution kernels	19
	Kernel size	3
BiGRU	Unidirectional hidden layer dimension	36
	Number of stacked layers	3
PINN	Weight coefficient γ of physical loss term	0.1
Training configuration	Learning rate	$3.17\times 10^{-3}$
	L_2_ regularization coefficient	$1\times 10^{-6}$

**Table 3:** Sensitivity analysis of the physical constraint weight γ.

γ	MRE	MAE	RMSE	R^2^
0.02	0.0355	0.3448	0.4077	0.6231
0.05	0.0247	0.2386	0.3240	0.7619
0.1	0.0124	0.1135	0.1856	0.9245
0.2	0.0533	0.5072	0.5624	0.2825
0.5	0.1252	1.3824	1.42	−3.5734

## Data Availability

The original contributions presented in this study are included in the article. Further inquiries can be directed to the corresponding authors.

## Abbreviations

The following abbreviations are used in this manuscript:

WOB	Weight on bit
DWOB	Downhole weight on bit
SWOB	Surface weight on bit
ROP	Rate of penetration
SPP	Standpipe pressure
MWD	Measurement while drilling
CNN	Convolutional neural network
GRU	Gated recurrent unit
BiGRU	Bidirectional gated recurrent unit
PINN	Physics-informed neural network
WOA	Whale optimization algorithm
WOA-PINN-CNN-BiGRU	Whale optimization algorithm-optimized physics-informed CNN-BiGRU model
RF	Random forest
SVM	Support vector machine
RNN	Recurrent neural network
LSTM	Long short-term memory
MRE	Mean relative error
MAE	Mean absolute error
RMSE	Root mean squared error
